# Histovariability and fossil diagenesis of *Pissarrachampsa* (Pseudosuchia, Notosuchia, Baurusuchidae) from the Upper Cretaceous of Southeast Brazil

**DOI:** 10.1002/ar.70021

**Published:** 2025-07-18

**Authors:** Tito Aureliano, Virgínia Maciel, Pedro Victor Buck, Felipe C. Montefeltro, Thiago da S. Marinho, Aline M. Ghilardi

**Affiliations:** ^1^ Department of Biological Chemistry, Programa de Pós‐Graduação em Diversidade Biológica e Recursos Naturais Regional University of Cariri (URCA) Crato Brazil; ^2^ Diversity, Ichnology and Osteohistology Research Group (DINOlab) Federal University of Rio Grande do Norte (UFRN) Natal Brazil; ^3^ Programa de Pós‐Graduação em Sistemática e Evolução, Centro de Biociências Federal University of Rio Grande do Norte (UFRN) Natal Brazil; ^4^ Laboratório de Paleontologia, Departamento de Ciências Exatas e da Terra Universidade do Estado de Minas Gerais (UEMG) Ituiutaba Brazil; ^5^ Laboratório de Paleontologia e Evolução de Ilha Solteira Universidade Estadual de São Paulo Ilha Solteira Brazil; ^6^ Centro de Pesquisas Paleontológicas “Llewellyn Ivor Price” Universidade Federal do Triângulo Mineiro Uberaba Brazil

**Keywords:** anatomy, Archosauria, paleontology, petrography, Pseudosuchia

## Abstract

Notosuchians were key components of western Gondwanan Cretaceous ecosystems in terrestrial predator niches and exhibited remarkable taxonomic and ecological diversity. Previous research has explored their physiology, metabolism, and histology, revealing varied growth patterns and life history strategies. While significant advancements have been made in recent years, there is much to unveil about the evolution of growth rate strategies within this clade. Here, we analyzed the histological variability of *Pissarrachampsa sera*, a baurusuchid from the Upper Cretaceous Adamantina Formation in Southeast Brazil, to investigate its growth dynamics and ecological adaptations. Thin sections from the femur, tibia, and fibula revealed fibrolamellar bone tissue with varied vascularization patterns, including radial, reticular, plexiform, laminar, and longitudinal canals. These patterns indicate differential growth rates among skeletal elements, with the tibia growing the fastest and the fibula the slowest. External Fundamental System and multiple Lines of Arrested Growth suggest somatic maturity in this young adult specimen. Limited diagenetic artifacts ensured reliable preservation for paleohistological interpretation. This study provides valuable information about notosuchian physiology and their evolutionary success in Gondwanan terrestrial ecosystems during the Mesozoic. Future investigations should aim to expand histological sampling across diverse taxa to refine our understanding of notosuchian growth strategies and ecological roles.

**Abbreviations**
DGM: Museu de Ciências da Terra, CPRM (Companhia de Pesquisa de Recursos Minerais); former Divisão de Geologia e Mineralogia of the Agência Nacional de Mineração, Rio de Janeiro, Brazil
IPS: Institut Català de Paleontologia Miquel Crusafont, Universitat Autònoma de Barcelona, Spain
MAU: Museo Municipal “Argentino Urquiza,” Puesto Hernandez, Argentina
MCT: Museu de Ciências da Terra—CPRM (Companhia de Pesquisa de Recursos Minerais; former Divisão de Geologia e Mineralogia of the Agência Nacional de Mineração, Rio de Janeiro, Brazil
MN; LPITB: Laboratory of Paleontology, Minas Gerais State University, Ituiutaba, Brazil
MPCA: Museo Provincial “Carlos Ameghino,” Cipolleti, Provincia de Río Negro, Argentina
MPMA: Museu Paleontológico de Monte Alto “Antônio Celso de Arruda Campos,” Monte Alto, Brazil

## 1 INTRODUCTION

Notosuchia (Leardi et al., 2024; Ruiz et al., 2021) represent a taxonomically and ecologically diverse clade of predominantly terrestrial crocodyliforms that achieved considerable evolutionary success during the Mesozoic, particularly throughout the Cretaceous of Gondwana, before experiencing gradual extinction through the Cenozoic until their ultimate disappearance in the Neogene (Filippi et al., 2013; Iori et al., 2024; Leardi et al., 2024; Nascimento & Zaher, 2010; Nicholl et al., 2021; Paolillo & Linares, 2007). Notosuchians reached their greatest taxonomic and morphological diversity in the Late Cretaceous continental ecosystems of South America, with the Bauru Group of Southeast Brazil serving as an exceptional repository of their evolutionary radiation (Bronzati et al., 2015; Filippi et al., 2013; Iori et al., 2024; Leardi et al., 2024; Nascimento & Zaher, 2010; Nicholl et al., 2021; Paolillo & Linares, 2007). Within these paleoecosystems, notosuchians demonstrably exceeded other predatory taxa, such as theropod dinosaurs, in both numerical abundance and taxonomic diversity, suggesting they occupied predominant roles in the trophic structure of these ancient communities (Andrade et al., 2023; Ghilardi, 2015; Montefeltro et al., 2020). Recent paleobiological investigations have increasingly focused on the histological characteristics of notosuchian remains, revealing variable growth dynamics ranging from relatively slow to moderate rates of skeletal development depending on the taxonomic affiliation (Navarro et al., 2025; Ricart et al., 2021). These histological signatures provide crucial insights into these extinct crocodyliforms' physiological capabilities and life history strategies (Cubo et al., 2020; de Andrade, 2018; Scheyer, 2025). Previous histological analyses have successfully documented growth patterns across multiple notosuchian lineages, including representatives of Uruguaysuchidae, Peirosauridae, Itasuchidae, and various sphagesaurian and sebesuchian groups (Andrade et al., 2023; Cubo et al., 2017; Fernández Dumont et al., 2021; Garcia Marsà et al., 2022; Marchetti et al., 2022; Navarro et al., 2025; Sena et al., 2018, 2022). Notably, within the phylogenetically diverse Sebecosuchia, only members of Sebecidae and Pissarrachampsinae lack comprehensive histological characterization, representing a gap in our understanding of notosuchian growth strategies (Navarro et al., 2025). The present investigation aims to elucidate the histological variability within *Pissarrachampsa* (Montefeltro et al., 2011), a baurusuchid crocodyliform recovered from the Santonian‐Campanian Adamantina Formation exposed in Minas Gerais state, Southeast Brazil. This research substantially enhances our understanding of notosuchian bone histology through detailed microstructural analysis of skeletal elements. It contributes valuable data toward a more nuanced understanding of the diverse growth strategies that evolved within this remarkable clade of Mesozoic crocodyliforms. Furthermore, this investigation provides additional context for interpreting the exceptional evolutionary success of notosuchians in Gondwanan terrestrial ecosystems during the Late Cretaceous Period.

## 2 MATERIALS AND METHODS

### 2.1 Specimen

LPITB‐PV 57 (Laboratory of Paleontology, Minas Gerais State University, Ituiutaba, Brazil) comprises a partial right femur, partial right tibia, partial right fibula, an almost complete right pes, and an almost complete forelimb, some cervical and caudal vertebrae, and a fragmentary skull and mandible, including part of the left maxilla, including the third maxillary tooth (hypertrophied), left jugal, partial left quadratojugal and quadrate, most of the left dentary and left surangular, left angular, and left partial articular of a baurusuchid notosuchian. As the fossils of LPITB‐PV 57 are of similar size and originate from the same monospecific locality and strata as all *Pissarrachampsa sera* specimens, we attribute them to the same taxon. Godoy et al. (2016) appended the diagnosis provided by Montefeltro et al. (2011), including postcranial diagnostic features for *P. sera*. Based on these two studies, we can observe the following diagnostic characters in LPITB‐PV 57: (1) baurusuchid with four maxillary teeth; (2) quadrate lateral depression with rostrocaudally directed major axis; (3) ulnar shaft subtriangular in cross‐section and strongly bowed laterally; (4) well‐developed femoral femorotibialis ridge; (5) complete absence of postcranial osteoderms.

The femur is broken at the middle of the diaphysis, with its distal portion missing and its proximal portion eroded. The tibia, like the femur, is also broken at the middle of the diaphysis and has its distal portion missing. The fibula is highly anteroposteriorly compressed, with its proximal, distal, and lateral portions missing.

### 2.2 Locality and horizon

The Adamantina Formation (Upper Cretaceous—Bauru Group) was originally defined by Soares et al. (1980) as comprising primarily fine to very fine reddish sandstones interbedded with siltstones and mudstones. The depositional environment was interpreted as a braided fluvial system with channel, sandbar, and lacustrine facies. Arai and Dias‐Brito (2023) proposed an Upper Santonian to Lower Campanian interval for the deposits of the Adamantina Formation. The only radiometric date for this unit (85.2 ± 2.7 Ma) was provided by Castro et al. (2018) for a fossiliferous site in General Salgado, western São Paulo state. In Minas Gerais state, the Adamantina Formation outcrops in several areas of the Triângulo Mineiro region and, in some localities, is overlain by the Upper Cretaceous Marília Formation (Batezelli, 2010).

The Campina Verde county is particularly important in the Triângulo Mineiro region, which has at least two fossiliferous sites yielding notosuchians, one of which has yielded all specimens of *P. sera* recovered to date. The outcrop of *P. sera* is located near the margins of BR‐364 (Montefeltro et al., 2011, figure 1). This locality is monospecific, with skeletal remains described so far attributed only to *P. sera* (Godoy et al., 2016, 2018; Montefeltro, 2019; Montefeltro et al., 2011). Marsola et al. (2016) described eggs with crocodilian affinity from this same locality of *P. sera* and tentatively attributed them to this taxon. Marsola et al. (2016) also conducted facies and pedological analyses, indicating the presence of a shallow lacustrine environment or playa lake with ephemeral channels and eolian deposition that contributed to the formation of a sandy soil.

### 2.3 Computed tomography scan

The femur, tibia, and fibula of LPITB‐PV 57 were scanned using a Philips Diamond Select Brilliance computed tomography (CT) 16‐slice medical scanner with over 200 slices each and a voxel size of 0.75 mm at the Hospital Universitário, Federal University of São Carlos (UFSCar), São Carlos city, Brazil. Acceleration voltage varied between 90 and 120 kV at a current of 367 mA. We used the workflow presented by Aureliano et al. (2020) to generate the three‐dimensional reconstruction of the elements in 3D Slicer v.5.6 (Fedorov et al., 2012). CloudCompare v.2 (Girardeau‐Montaut, 2016) generated realistic gray‐shaded photographs of these digital models with an ambient occlusion filter.

### 2.4 Paleohistology

We extracted samples from the femur, tibia, and fibula midshaft cross‐sections to describe bone microstructure. The samples were removed using a Dremel® electric saw. Polished sections were then stabilized with PaleoBOND® PB0020 and embedded into Araldite® 2020 resin. Thereafter, samples were ground to a thickness of approximately 50–60 μm. We analyzed and photographed the thin sections on an Olympus BX53‐P polarized microscope attached to an Olympus U‐TV0.5XC‐3 at the Seismology Laboratory (LabSis, UFRN). We followed Francillon‐Viellot et al. (1990) and Huttenlocker et al. (2013) for histological descriptions. We also evaluated the diagenetic aspects of LPITB‐PV 57, including petrography and fracturing.

## 3 RESULTS

### 3.1 Computed tomography scan

**FIGURE 1:**
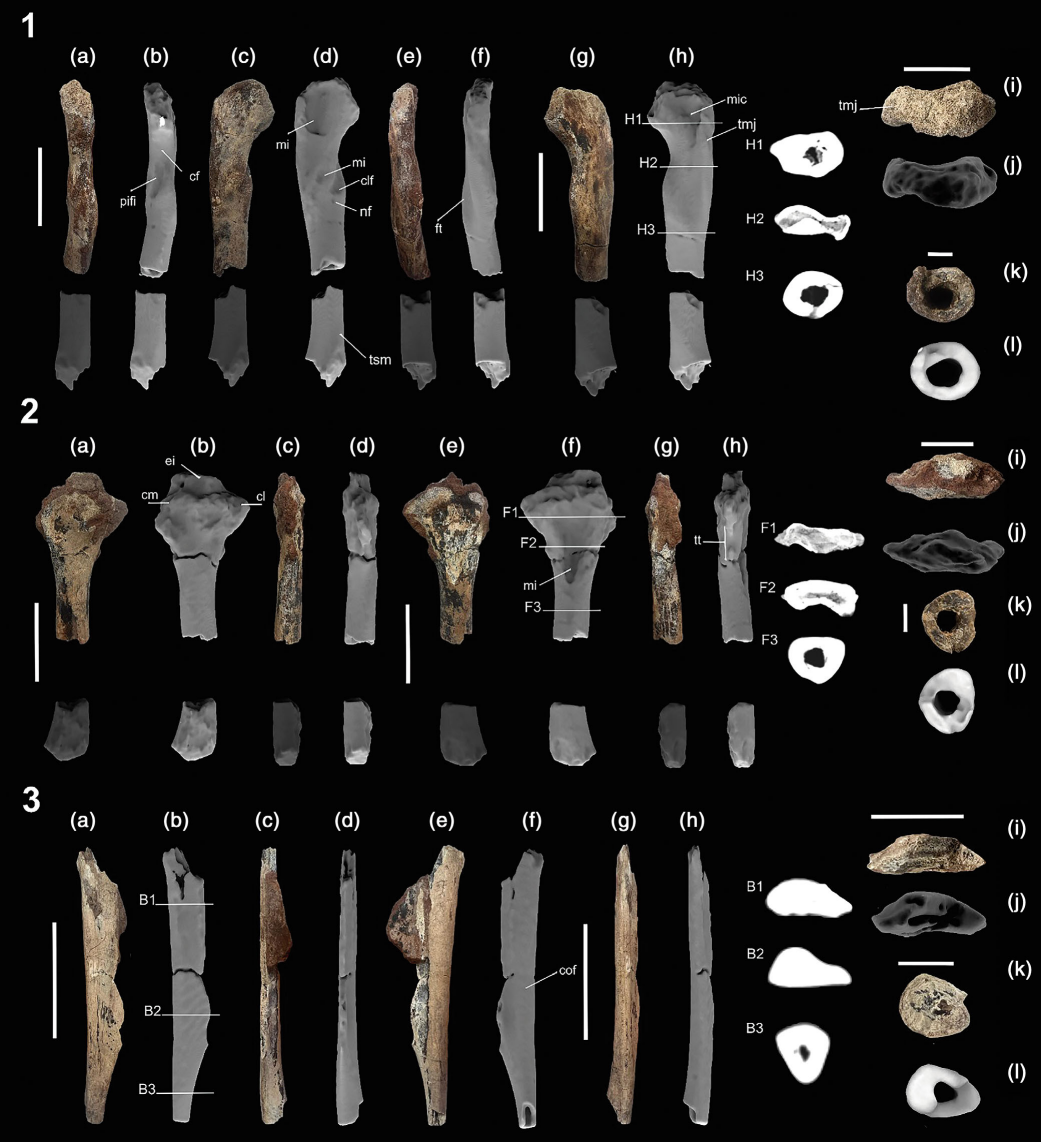
*Pissarrachampsa* LPITB‐PV 57, photographs (a, c, e, g, i, k) and 3D models created from computed tomography (CT) scan (b, d, f, h, j, l). (1) *Left femur* in anterior (a, b), medial (c, d), posterior (e, f), lateral (g, h), proximal (i, j) and distal (k, l) views. (h1–h3), Cross‐sections of the CT scan (shown in 1h). (2) *Right tibia* in anterior (a, b), medial (c, d), posterior (e, f), lateral (g, h), proximal (i, j), and distal (k, l) views. (f1–f3) Cross‐sections of the CT scan (shown in 2f). (3) *Right fibula* in anterior (a, b), lateral (c, d), posterior (e, f), medial (g, h), proximal (i, j), and distal (k, l) views. (b1–b3) Cross‐sections of the CT scan (shown in 3b). cf, corpus femoris; cl, condylus lateralis; clf, *Musculus caudofemoralis longus*; cm, condylus medialis; cof, corpus fibulae; ei, eminentia intercondylaris; ft, fourth trochanter; mi, muscular insertion; mic, *M. ischiotrochantericus*; nf, nutrient foramen; pifi, *M. puboischiofemoralis internus*; tmj, trochanter major; tsm, tuberositas supracondylaris medialis; tt, tuberositas tibiae. Scale in (a–h) = 50 mm; (i and j) = 20 mm, (k and l) = 10 mm. h1–h3, f1–f3, b1–b3 = not to scale.

The cortex of the three specimens decreases toward the epiphyses. There is the presence of trabecular bone tissue, mainly proximally toward the diaphysis in the femur and tibia (Figure 1h1–h3, f1–f3). All specimens are highly worn. The left femur shows wear on the proximal view, with total rupture in the diaphysis and the distal epiphysis (Figure 1; 1a–h). Similar to the femur, the right tibia shows wear on the proximal view and ruptures in the diaphysis and distal epiphysis. Additionally, there is a fracture resulting from taphonomic processes visible in all views (Figure 1; 2a–h). The right fibula shows a prominent accumulation of sediment in the diaphysis and two large fractures, mainly visible in the anterior view (Figure 1; 3a,b). Furthermore, both the proximal and distal epiphyses are incomplete.

### 3.2 Osteohistological description

**FIGURE 2:**
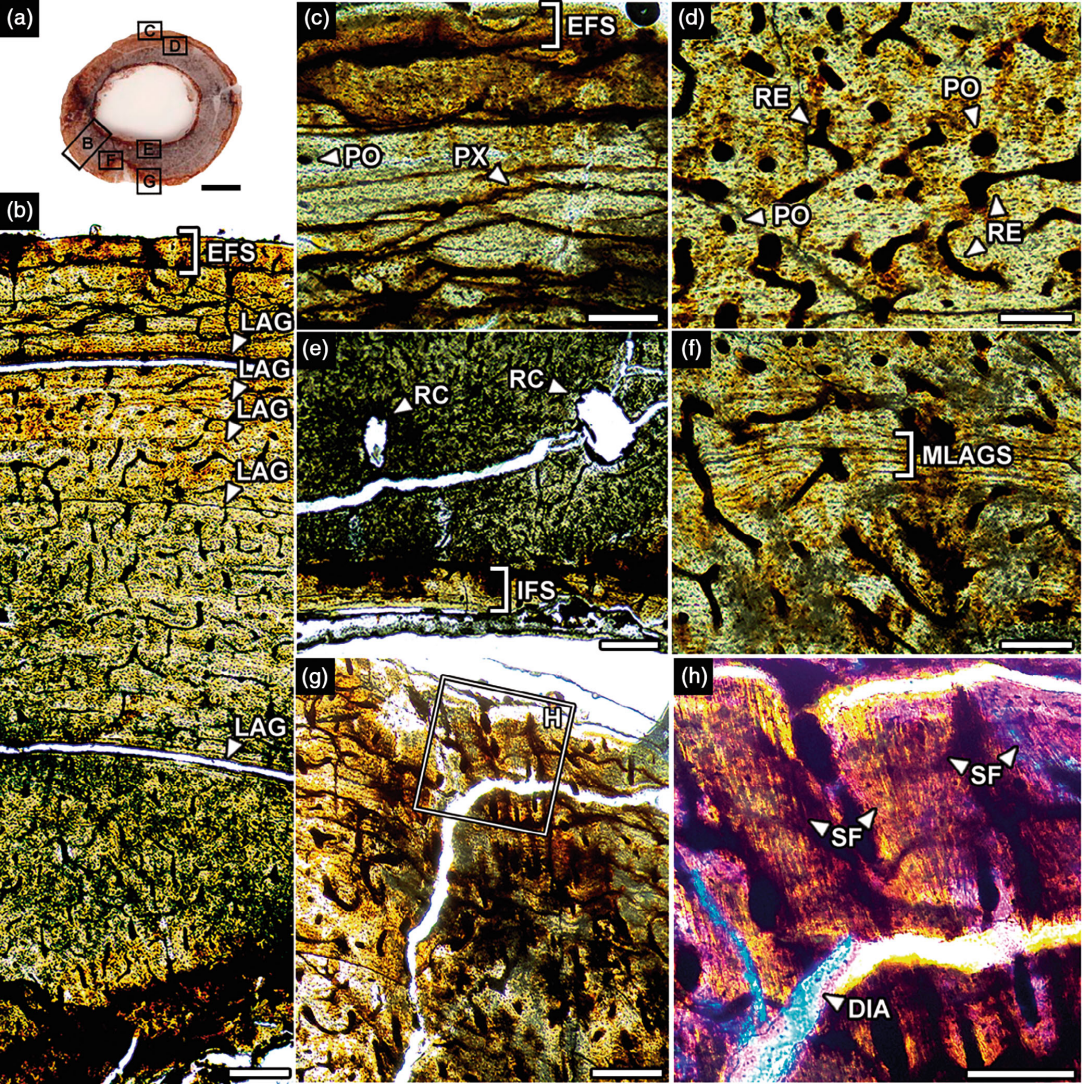
Femur histology of the baurusuchid notosuchian *Pissarrachampsa* LPITB‐PV 57 from the Upper Cretaceous of Southeast Brazil. (a) Midshaft cross‐section showing the index of areas detailed in (b–h). (b) histologic profile. DIA, diagenetic artifact; EFS, External Fundamental System; IFS, Internal Fundamental System; LAG, Line of Arrested Growth; MLAGS, multiple LAGs; PO, primary osteon; PX, plexiform canal; RC, resorption cavity; RE, reticular canal; SF, Sharpey's fibers. Normal light in (a). Polarized under parallel nicols in (c, g). Crossed nicols with lambda compensator in (h). Scale bar in A = 5 mm; (b, e, g) = 200 μm; (c, d, f, h) = 100 μm.

The entire cortex of the femur comprises primary bone tissue (fibrolamellar, parallel‐fibered, and, to a lesser extent, lamellar bone; see Figure 2). Vascularization presents chaotic angles varying from reticular to (predominantly) plexiform patterns from the inner to the outer cortex, respectively. An External Fundamental System (EFS) is present. Additionally, an Internal Fundamental System (IFS) is also present. Laminar vascularization is also present and contains some (at least five) Lines of Arrested Growth (LAGs) with increasing spacing toward the periosteum. There are multiple (double or triple) LAGs in the middle cortex (Figure 2f). Some primary osteons exist, but more commonly in the outer cortex. Resorption cavities are in the inner cortex (Figure 2e). The medullary cavity is free of any cancellous bone tissue. Sharpey's fibers extend deeply and medially at high angles (Figure 2g,h). These fibers (Figure 2h) show high optical relief and birefringence (Aureliano, Ghilardi, Fernandes, et al., 2023).

**FIGURE 3:**
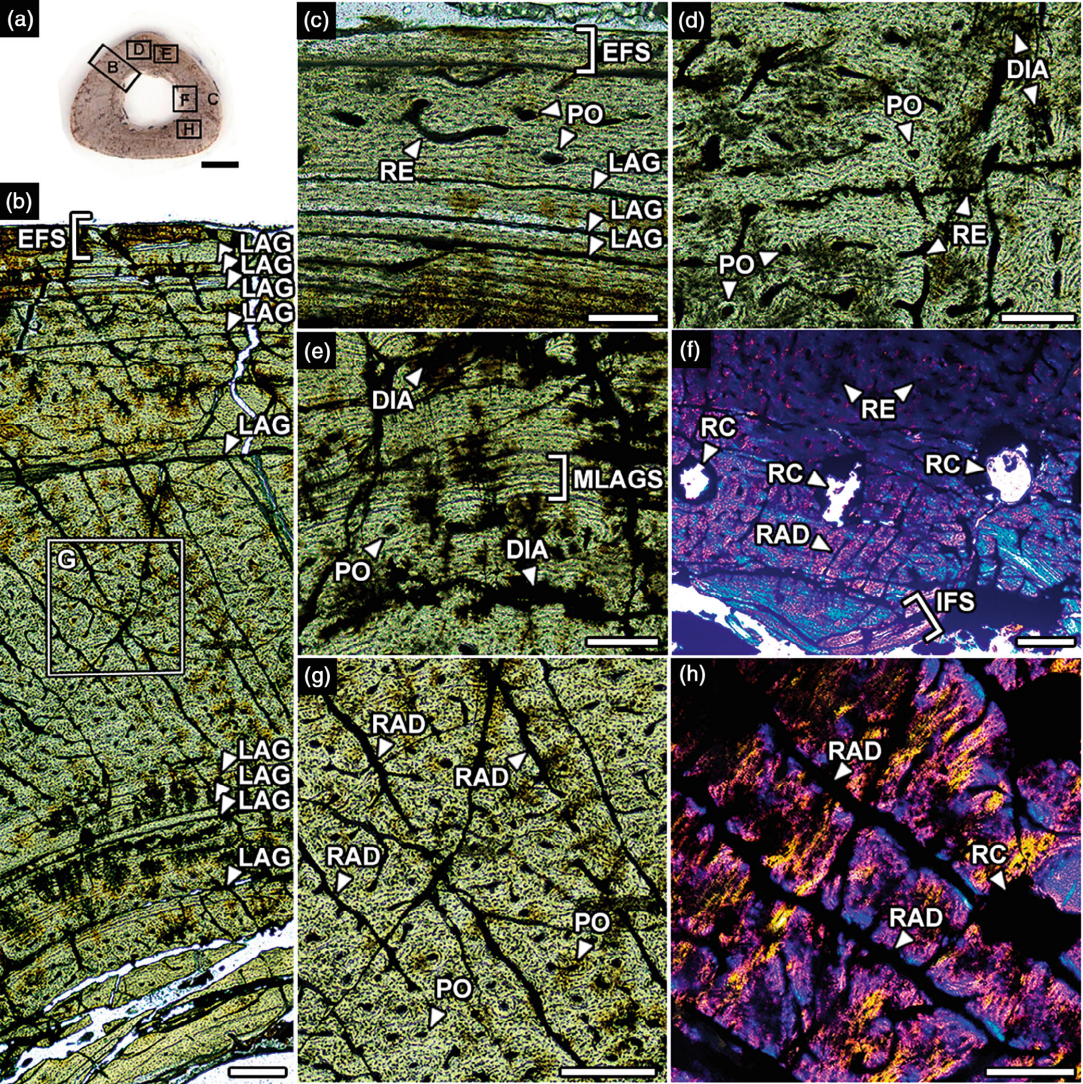
Tibia histology of the baurusuchid notosuchian *Pissarrachampsa* LPITB‐PV 57 from the Upper Cretaceous of Southeast Brazil. (a) Midshaft cross‐section showing the index of areas detailed in (b–h). (b) Histologic profile. DIA, diagenetic artifacts; EFS, External Fundamental System; IFS, Internal Fundamental System; LAG, Line of Arrested Growth; MLAGS, multiple LAGs; PO, primary osteon; RAD, radial canal; RC, resorption cavity; RE, reticular canal. Normal light in (a). Polarized under parallel nicols in (b–e, g). Crossed nicols with lambda compensator in (f, h). Scale bar in (a) = 5 mm; (b, f, g) = 200 μm; (c–e, h) = 100 μm.

The thin section of the tibia reveals that the cortical microstructure comprises primary tissue (Figure 3b). Longitudinal tissue (primary osteons) dominates the vascularization in the inner to middle cortex and is also present in the outer cortex sparsely (Figure 3b–e,g). Laminar vascularization predominates from the middle to the outer cortex with over nine LAGs. However, LAGs are absent in the middle cortex and concentrate in the inner and outer extremes (Figure 3b). There are multiple (triple) LAGs in the middle cortex (Figure 3e). An EFS is present across the periosteal perimeter. There are large radial canals across the cortex (Figure 3g,h). There are resorption cavities in the inner cortex (Figure 3f,h) and an IFS deposited in the perimedullary region (Figure 3f).

**FIGURE 4:**
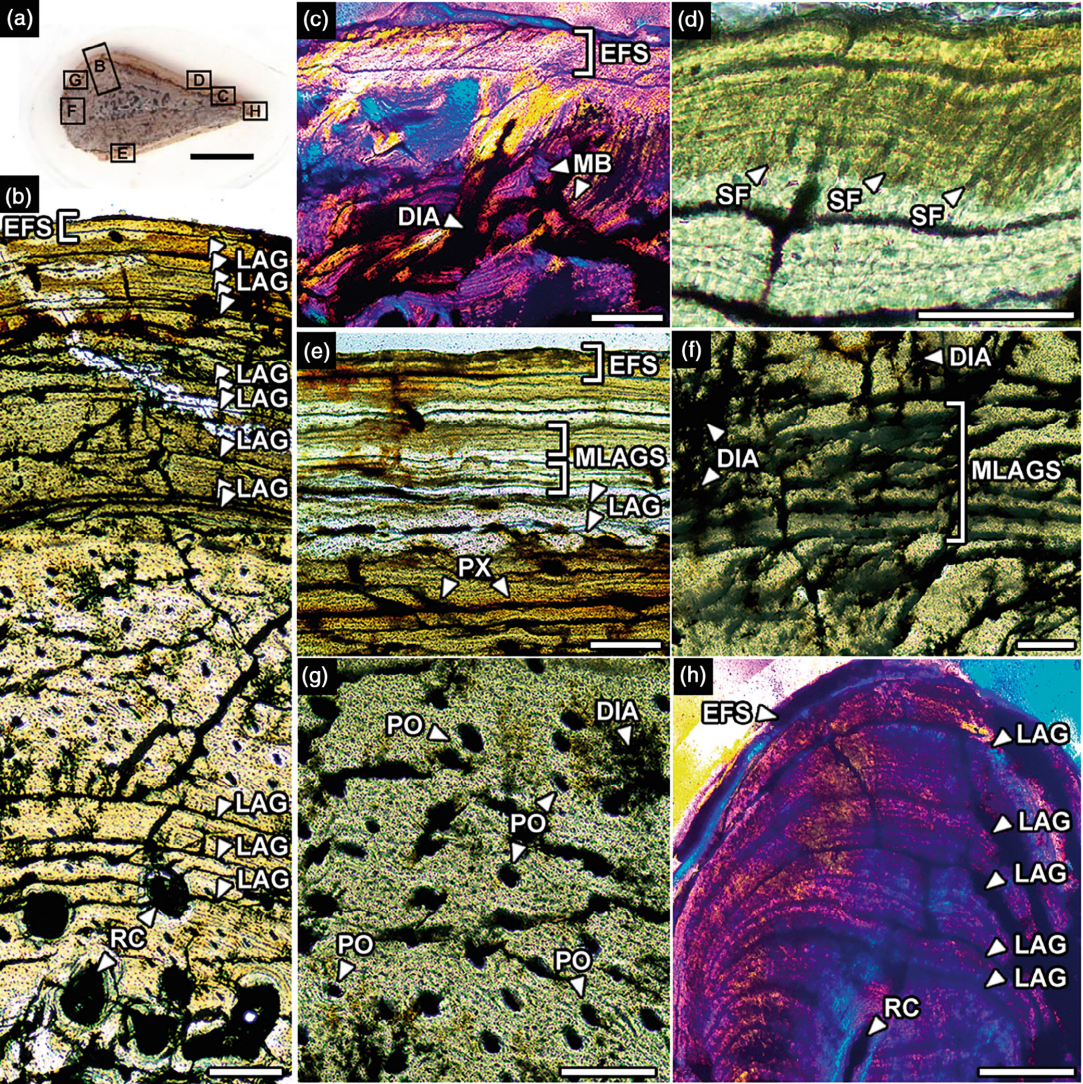
Fibula histology of the baurusuchid notosuchian *Pissarrachampsa* LPITB‐PV 57 from the Upper Cretaceous of Southeast Brazil. (a) Midshaft cross‐section showing the index of areas detailed in (b–h). (b) Histologic profile. DIA, diagenetic artifacts; EFS, External Fundamental System; IFS, Internal Fundamental System; LAG, Line of Arrested Growth; MB, Medullary Bone; MLAGS, multiple LAGs; PO, primary osteon; PX, plexiform canal; RC, resorption cavity; SF, Sharpey's fibers. Normal light in (a). Polarized under parallel nicols in (c, g). Crossed nicols with lambda compensator in (h). Scale bar in (a) = 5 mm; (b) = 200 μm; (c, e–h) = 100 μm; (d) = 50 μm.

The entire cortex of the fibula also comprehends primary bone tissue (Figure 4). However, most of the tissue is organized longitudinally, with primary osteons ranging from the endosteum to the outer cortex. Laminar vascularization is also present, with over 10 LAGs concentrated in the inner and outer cortex and absent in the middle portion, where primary osteons abound (Figure 4b). There are multiple (sixtripple) LAGs in the middle cortex (Figure 4e,f). The medullary cavity is filled with bone. Sharpey's fibers are present in the periosteal area at acute angles (Figure 4c,d), presenting high optical relief and birefringence (Aureliano, Ghilardi, Fernandes, et al., 2023; Petermann & Sander, 2013). There is a shortening of the cortex laterally, and the EFS neighbors the medullary region in this specific spot (Figure 4c). There are resorption cavities in the inner cortex (Figure 4b).

The presence of an EFS on both the femur, tibia, and fibula suggests that the individual was an adult at the time of death (Francillon‐Viellot et al., 1990). However, the predominance of fibrolamellar tissue composed of plexiform, reticular, and radial vascularizations, plus the presence of resorption cavities, suggests a young adult status (Chinsamy‐Turan, 2005; Francillon‐Viellot et al., 1990; Huttenlocker et al., 2013; Klein & Sander, 2008; Mitchell & Sander, 2014).

### 3.3 Diagenetic artifacts

There are limited macroscopic breaks and no signs of lithostatic compression. Abrasion traces are almost absent. The thin sections are moderately well preserved and show few diagenetic artifacts. There are cracks both following pre‐existing LAGs (Figure 2b) and running randomly across the cortex (Figures 2g,h and 4b,f). There are signs of precipitating opaque minerals following these cracks and also filling pores from vascular canals and erosion chambers (Figures 3h and 4b).

## 4 DISCUSSION

The cross‐section of the femur, tibia, and fibula of *Pissarrachampsa* LPITB‐PV 57 showed similar ontogenetic signatures but were actively growing at different rates. Different vascularization types indicate the rate of tissue deposition (Chinsamy‐Turan, 2005; Huttenlocker et al., 2013). Primary tissue decreases in deposition rate according to the amount of blood proportional to the bone, decreasing in the following order: radial → reticular → plexiform → laminar → longitudinal (Francillon‐Viellot et al., 1990). The presence of radial canals rendered the tibia the highest growth rate, while the fibula presented the slowest rate, with the most laminar and longitudinal vascularization (de Margerie et al., 2002, 2004). Differences in growth rates in distinct appendicular elements within the same individual have been reported for other vertebrates, including mammals, dinosaurs, mesoeucrocodylians, and others (Eugenia et al., 2025; Padian et al., 2016; Starck & Chinsamy, 2002). This dynamic appears taxon‐specific and related to allometry (Starck & Chinsamy, 2002; Woodward et al., 2011). Morphological analyses of notosuchians reveal distinct limb proportion adaptations linked to cursorial locomotion. Comparative studies of *Caipirasuchus* MPMA 07‐0011/00 (Museu Paleontológico de Monte Alto “Antônio Celso de Arruda Campos,” Monte Alto, Brazil) demonstrate a zeugopodium/stylopodium ratio of 1.8:1 in the hindlimbs, with the tibia contributing 55% of total limb length—a configuration interpreted as enhancing agility and predator evasion (Iori & Carvalho, 2011). This pattern is corroborated in *Baurusuchus albertoi*, which, despite having a zeugopodium/stylopodium hindlimb ratio of 0.7:1, the sacral vertebrae exhibit dorsolaterally deflected transverse processes (15°–20° inclination) that biomechanically facilitate increased stride length during rapid locomotion (Nascimento & Zaher, 2010). The consistent elongation of posterior zeugopodia across these taxa suggests strong selective pressures for cursorial efficiency in Late Cretaceous ecosystems, potentially related to niche competition with contemporaneous theropods (Andrade et al., 2023). Consequently, tibiae had to develop faster to achieve larger sizes in proportion to the femora.

### 4.1 Remarks on notosuchian growth strategies

An EFS in notosuchians has only been reported for *Iberosuchus* IPS 4930 (Institut Català de Paleontologia Miquel Crusafont, Universitat Autònoma de Barcelona, Spain) (Cubo et al., 2017), *Pepesuchus* MN 7466‐V (Sena et al., 2018), and *Pissarrachampsa* LPITB‐PV 57. This could mean that either all sampled notosuchians were somatically immature or the selected skeletal elements of these specimens did not preserve EFS.

Notosuchians had diverse sizes and growth patterns among clades, rendering it impractical to encapsulate their growth strategies within a unified framework. Growth rates vary from low in the femur and humerus of the uruguaysuchid *Araripesuchus* MPCA PV 242 (Museo Provincial “Carlos Ameghino,” Cipolleti, Provincia de Río Negro, Argentina) (Fernández Dumont et al., 2021) to middle in the tibia and fibula of the peirosaurid MAU‐PV‐PH 437 (Museo Municipal “Argentino Urquiza,” Puesto Hernandez, Argentina) (Navarro et al., 2025), and high rates in the ulna and tibia of *Pepesuchus* MN 7466‐V (Sena et al., 2018), both itasuchids, and in the femur, humerus, and ulna of *Notosuchus* MPCA‐250 (Navarro et al., 2023). These differences in growth rates do not correlate with body size in notosuchians, since larger taxa such as *Iberosuchus* could present slow growth rates similar to those of the small uruguaysuchids. This condition might not be strictly linked to phylogenetic proximity, given the variability present in the itasuchids mentioned. However, baurusuchids such as *Pissarrachampsa* LPITB‐PV 57, *Stratiotosuchus* DGM 1477‐R (Museu de Ciências da Terra, CPRM [Companhia de Pesquisa de Recursos Minerais]; former Divisão de Geologia e Mineralogia of the Agência Nacional de Mineração, Rio de Janeiro, Brazil), and MCT 1714‐R (Museu de Ciências da Terra—CPRM [Companhia de Pesquisa de Recursos Minerais]; former Divisão de Geologia e Mineralogia of the Agência Nacional de Mineração, Rio de Janeiro, Brazil) (Andrade et al., 2023), and *Baurusuchus* MPMA 62.0002.02 (Marchetti et al., 2022) all present high growth rates. The extensive radial vascular canals observed in *Pissarrachampsa* LPITB‐PV 57 likewise indicate elevated rates of bone deposition. Such features are posited to develop in response to significant nutritional requirements in large‐bodied taxa exhibiting rapid somatic growth (Chinsamy et al., 2020; Nacarino‐Meneses & Chinsamy, 2022). Other factors might influence this variation in taxon body size and growth dynamics, and environmental pressure (e.g., resource availability and ecological competition) cannot be discarded; its effects on growth strategies have been described in other archosaurian lineages within the Bauru Group units (Aureliano, Ghilardi, Fonseca, et al., 2023; Navarro et al., 2022). Finally, our histovariability analysis showed that different appendicular elements presented distinct growth signatures. This mechanism should be better studied in other taxa to improve control of this variable, since histological comparisons in Notosuchia often rely on distinct skeletal elements and even on mesochondral samples (osteoderms).

### 4.2 Remarks on the diagenetic features of fossils from the Bauru Group

Fossils from units within the Bauru Group present variable degrees of microstructural preservation directly linked to specific sedimentary contexts and morphological and ecological particularities of organisms (Aureliano et al., 2020, 2021; Aureliano, Ghilardi, Fernandes, et al., 2023; Aureliano, Ghilardi, Fonseca, et al., 2023; Azevedo et al., 2013; Marchetti et al., 2019; Paio et al., 2024). Regarding fossil diagenesis, specimens from fluvial deposits of the Adamantina and São José do Rio Preto formations are better preserved. Fossils within these units are affected by diminutive microfracturing, with eo and telodiagenetic artifacts represented by siliclastic grain infilling and opaque minerals, respectively (Aureliano et al., 2021; Aureliano, Ghilardi, Fernandes, et al., 2023; Marchetti et al., 2019). Skeletal remains from the distributive fluvial system of the Serra da Galga Formation, affected by calcretization in some areas, may preserve well 3D shapes but are impacted by calcite‐infilled cracks, often destroying histological information (Aureliano et al., 2020; Aureliano, Ghilardi, Fonseca, et al., 2023). Finally, fossils from the Marília Formation could have been affected by strong eodiagenetic markers such as transportation fractures, silicate mineral and pyrite infilling, and calcite precipitation (Paio et al., 2024).

The skeletal morphology of crocodyliforms presents overall thicker compact cortices and tight trabecular arrays when compared to saurischians, especially theropods, in both appendicular and axial elements (Aureliano et al., 2018, 2022; Fabbri et al., 2022). This microstructural adaptation might have favored the preservation of notosuchian fossils compared to saurischians. Niche partitioning might also have favored the exceptional microstructural preservation of articulated notosuchian specimens found in the Adamantina Formation (Bandeira et al., 2018; Ghilardi, 2015).

Our analysis of *Pissarrachampsa* limb elements from the Adamantina Formation reveals a notably moderate degree of diagenetic alteration. Unlike many Bauru Group fossils that exhibit extensive lithostatic compression and pervasive mineral replacement, the studied specimens show only limited macroscopic breaks, minimal abrasion, and well‐preserved cortical microstructure. Cracks observed in thin section frequently follow pre‐existing LAGs or cross the cortex randomly, with opaque mineral precipitates infilling both cracks and vascular canals. This pattern of diagenetic modification is less severe than that reported in other Bauru Group taxa, suggesting localized variation in burial and fossilization conditions. Importantly, the preservation quality in our material enables detailed histological analysis, providing new insights into notosuchian bone growth that might be obscured in more heavily altered specimens. These findings contribute to a refined understanding of the diagenetic spectrum present in the Bauru Group and underscore the importance of site‐specific taphonomic histories for histological studies.

## 5 CONCLUSIONS

This study presents a comprehensive histological analysis of *P. sera*, a baurusuchid notosuchian from the Upper Cretaceous Adamantina Formation in Southeast Brazil. The findings reveal minor histovariability across the femur, tibia, and fibula, offering a detailed understanding of the growth dynamics of this taxon. The presence of fibrolamellar bone tissue, varying vascularization patterns, and EFS suggests that the individual was a young adult at the time of death. These observations enhance our understanding of notosuchian growth strategies and their ecological adaptations. Our main highlights include:

1. Histological analysis confirmed ceased growth in *Pissarrachampsa* across different skeletal elements, with distinct vascularization patterns indicating variable growth rates.
2. The tibia exhibited the fastest growth rate due to radial vascularization, while the fibula showed slower growth characterized by laminar and longitudinal vascularization.
3. The presence of EFS and multiple LAGs indicates somatic maturity and ceased growth in this young adult specimen.
4. Limited diagenetic artifacts ensured reliable preservation of microstructural details for paleohistological interpretation.

These results demonstrate the evolutionary success of notosuchians in Gondwanan ecosystems during the Late Cretaceous, emphasizing some of their unique physiological strategies. Specifically, the histology of *Pissarrachampsa* reveals a combination of rapid and sustained limb growth, skeletal maturity, and adaptive limb specialization, which likely contributed to notosuchian ecological dominance and diversity in these ancient environments. Future research should further focus on expanding histological sampling across additional taxa and skeletal elements to elucidate growth patterns and ecological roles within this clade.

## AUTHOR CONTRIBUTIONS

**Tito Aureliano:** Conceptualization; investigation; funding acquisition; writing – original draft; methodology; validation; visualization; writing – review and editing; software; formal analysis; project administration; supervision. **Virgínia Maciel:** Investigation; writing – original draft; writing – review and editing; visualization; methodology; validation; software; formal analysis. **Pedro Victor Buck:** Data curation; supervision; resources; conceptualization; investigation; writing – original draft; writing – review and editing; validation; formal analysis. **Felipe C. Montefeltro:** Investigation; writing – original draft; writing – review and editing; validation; formal analysis; supervision. **Thiago da S. Marinho:** Validation; formal analysis; writing – original draft; writing – review and editing; supervision. **Aline M. Ghilardi:** Investigation; validation; formal analysis; writing – original draft; writing – review and editing; supervision.

## Data Availability

The specimen is housed in a public research institution (UEMG, Ituiutaba campus) and can be accessed upon request. CT scans are available on Morphosource (links provided in Section 2).
